# Trajectories and predictors of psychological distress during the first 6 months of weight loss among adults with obesity: a prospective longitudinal cohort study

**DOI:** 10.3389/fpsyt.2026.1883542

**Published:** 2026-09-30

**Authors:** Zhiqiang Cheng, Jiaoyun Xia, Jincheng Liu, Baozhen Zhang, Jinming Liu

**Affiliations:** 1 Department of Nursing, The First Affiliated Hospital of Nanchang University, Nanchang, China; 2 School of Nursing, Jiangxi Medical College, Nanchang University, Nanchang, China; 3 The First Clinical Medical College, Gannan Medical University, Ganzhou, China; 4 Department of Cardiovascular Internal Medicine, The First Affiliated Hospital of Gannan Medical University, Ganzhou, China

**Keywords:** obesity, psychological distress, risk factors, trajectory, weight loss

## Abstract

**Objectives:**

To characterize longitudinal trajectories of psychological distress from first presentation to a weight management clinic through 6 months of weight loss.

**Methods:**

Adults with obesity (body mass index, BMI ≥28 kg/m²) were consecutively recruited from October 2024 to May 2025. Psychological distress was evaluated at four time points: initial clinic presentation (T1), 1 month (T2), 3 months (T3), and 6 months (T4) after weight loss initiation. Latent class growth modeling (LCGM) was applied to identify heterogeneous psychological distress trajectories. Multinomial logistic regression was used to explore baseline predictors for trajectory membership, with the persistently no/low distress group as the reference group (C3).

**Results:**

LCGM identified three distinct longitudinal trajectories of psychological distress: Trajectory 1 (C1, 63.7%): gradually decreasing distress over 6 month s; Trajectory 2 (C2, 21.1%): continuously increasing distress; Trajectory 3 (C3, 15.2%): persistently no or low-level distress. Multinomial regression results showed that C1 membership was independently associated with younger age (OR=0.858, 95%CI: 0.745 to 0.988), history of chronic disease (OR = 1.293, 95%CI: 1.018 to 1.642), higher negative coping (OR = 1.445, 95%CI: 1.166 to 1.791), lower perceived social support (OR = 0.912, 95%CI: 0.832 to 1.000), and more negative illness perception (OR = 1.064, 95%CI: 1.002 to 1.163). C2 membership was independently associated with younger age (OR = 0.827, 95%CI: 0.760 to 0. 988), female sex (male vs female OR = 0.131, 95%CI: 0.024 to 0.708), higher BMI (OR = 1.337, 95%CI: 1.055 to 1.695), higher negative coping (OR = 1.319, 9 5%CI: 1.043 to 1.667), lower perceived social support (OR = 0.867, 95%CI:0.79–3 to 0.947), and more negative illness perception (OR = 1.080,95%CI:1.002 to 1.164).

**Conclusions:**

Psychological distress among obese adults during early weight loss presents obvious heterogeneity. Nearly one-fifth of patients experience progressive elevation in distress, which requires close clinical attention. Routine assessment of baseline psychosocial and clinical indicators can support early risk stratification and facilitate delivery of timely psychological and behavioral interventions within the first 6 months of weight loss.

## 1 Introduction

Obesity is a growing global epidemic. The World Health Organization (WHO) projects that by 2035, more than 4 billion people worldwide will be overweight or obese, accounting for about 51% of the global population. Obesity is an established independent risk factor for cardiovascular and cerebrovascular diseases, type 2 diabetes, and chronic kidney disease (1). It has become a leading contributor to death and disability, with substantial reductions in life expectancy and rapidly increasing healthcare expenditure attributable to obesity and its complications (2).

Beyond cardiometabolic consequences, obesity is increasingly recognized as a condition with a substantial psychological burden. During long-term weight management—characterized by progressive course, recurrent weight regain, and high demands for sustained lifestyle change—adults with obesity frequently experience psychological distress, including anxiety, depression, irritability, and mood instability (2). Psychological distress represents a continuum from vulnerability, sadness, and fear to more severe states such as depression, anxiety, panic, social isolation, and crisis, and has been proposed as an additional “vital sign” alongside pain in clinical assessment (3).

The burden of distress among obese individuals is not uniform. Social acceleration and increasing work-related stress may further heighten vulnerability to negative emotions among adults, and accumulating evidence suggests that tension, anxiety, and irritability may be linked to rising obesity rates (4). Cross-sectional data indicate that anxiety and depression are common in obese populations (5), and negative affect may exacerbate physical inactivity and worsen metabolic and neurovascular function through inflammatory and neuroendocrine pathways (6). In addition, body- image related stigma and obesity-related physical changes and complications may contribute to self-blame and depressive symptoms, which can further aggravate disordered eating and weight gain (7).

However, current evidence remains limited in a clinically critical way: most studies assess psychological status at a single time point, which cannot characterize heterogeneity in the longitudinal course of distress during weight loss. Because patients differ in age, sex, BMI, comorbidities, and their lived experiences during the weight- loss process, distress may follow distinct patterns (e.g., improving, worsening, or persistently low), but these latent subgroups cannot be reliably identified in cross- sectional analyses (4). This gap matters for clinical practice. Weight loss treatment usually aims to achieve a weight reduction of 5% to 15% within the first 6 months of early weight management. The implementation methods include lifestyle intervention, medication treatment, and bariatric surgery (8). Although these approaches can improve physical outcomes and may reduce distress in some patients (9).

Therefore, focusing on adults with obesity, this longitudinal study aims to (1) identify distinct latent trajectories of psychological distress during the weight-loss process using latent class growth modeling (LCGM), and (2) provide evidence to support early risk stratification and targeted psychological interventions for patients who are most likely to experience adverse distress trajectories.

## 2 Methods

### 2.1 Study design and participants

A prospective longitudinal cohort study was designed to characterize trajectories of psychological distress during early weight loss. Participants were consecutively recruited between October 2024 and May 2025 from the weight management center of a tertiary hospital in Jiangxi Province, China. Recruitment took place at The First Affiliated Hospital of Nanchang University, a large provincial tertiary referral hospital with multiple campuses and a high clinical throughput (e.g., >2.7 million outpatient visits in 2018), and substantial inpatient capacity across campuses (e.g., 2,900 beds at the headquarters and 3,200 beds at the Xianghu branch). The hospital therefore serves as a major referral center within Jiangxi Province (located in central China) and receives patients across a broad catchment area, which supports the pragmatic representativeness of this clinic-based cohort for the clinical profile of adults seeking obesity care in central China.

Eligible participants met all of the following inclusion criteria: (1) age 18 to 59 years; (2) BMI ≥ 28 kg/m²; (3) waist circumference ≥ 90 cm (men) or ≥ 85 cm (women); and (4) sufficient literacy to understand the questionnaires. Exclusion criteria were: (1) history of mental illness; (2) severe psychological trauma within the previous 3 months (e.g., bereavement or major family upheaval); or (3) concurrent severe cardiovascular/cerebrovascular disease or malignant tumors.

### 2.2 Sample size

The sample size required was calculated using the G*Power 3.1 software, With α = 0.05, 1-β =0.8, the expected effect size f=0.25, and measurements conducted 4 times (10), the required sample size was determined to be 160 cases. Considering a 20% dropout rate, the minimum sample size required for this study was 196 cases. The final sample size included in this study exceeded 200, thus meeting the minimum sample size requirement of this research.

### 2.3 Survey tools

#### 2.3.1 General information questionnaire

Sociodemographic and clinical characteristics were collected using a structured questionnaire developed by the research team based on relevant literature. Variables included sex, age, educational attainment, marital status, occupation, living arrangement, payment method for medical expenses, body mass index (BMI), body weight, waist circumference, weight-loss goal, history of chronic disease, and average monthly income.

#### 2.3.2 Distress thermometer

Psychological distress over the past week was assessed using the Distress Thermometer (DT), a widely used single-item visual rating scale ranging from 0 to 10, where higher scores indicate greater distress. Scores were interpreted as 0 (no distress), 1 to 3 (mild), 4 to 6 (moderate), 7 to 9 (severe), and 10 (extreme). A cut-off of DT ≥4 was used to define clinically relevant distress. The reported Cronbach’s α=0.914 in this study was calculated from combined data of all repeated DT measurements across four time points, reflecting the test-retest reliability of the DT in this cohort, rather than internal consistency of scale items (11).

#### 2.3.3 Perceived social support scale

Perceived social support was measured using the Perceived Social Support Scale (PSSS) (12), which includes 12 items across three domains: family, friends, and significant others/other support. Items are rated on a 7-point Likert scale (1=strongly disagree to 7=strongly agree), yielding total scores from 12 to 84; higher scores indicate greater perceived support. Scores were categorized as low (12 to 36), moderate (37 to 60), and high (61 to 84). The Cronbach’s α in this study was 0.932.

#### 2.3.4 Brief illness perception questionnaire

Illness perception was assessed using the Brief Illness Perception Questionnaire (BIPQ) (13), comprising 9 items covering cognitive, emotional, and understanding dimensions (including one open-ended item). Each item is rated from 0 to 10.The total score ranges from 0 to 80, with higher scores indicating more negative illness perceptions (i.e., stronger perceived threat). The reported reliability of the instrument is acceptable (Cronbach’s α=0.83) (14).

#### 2.3.5 Simplified coping style questionnaire

Coping style was measured using the Simplified Coping Style Questionnaire (SCSQ) (15), which assesses coping responses to illness across two dimensions: positive coping and negative coping. The SCSQ contains 20 items scored on a 4-point Likert scale (0 to 3). Dimension scores range from 0 to 36 (positive coping) and 0 to 24 (negative coping), with higher scores indicating greater use of the corresponding coping style. The Cronbach’s α in this study was 0.886.

### 2.4 Ethical review

The study protocol was reviewed and approved by the Ethics Committee of the hospital (approval No. IIT-2025-055). All participants provided written informed consent before enrolment. Participant confidentiality was safeguarded throughout the study; data were de-identified at collection, stored on password-protected systems with restricted access, and used solely for research purposes. The study was conducted in accordance with the principles of the Declaration of Helsinki and relevant local regulations.

### 2.5 Data collection

Data were collected at four time points: baseline (at the initial clinic visit) and at 1, 3, and 6 months after initiation of the weight-loss program. At baseline, participants completed a structured questionnaire on sociodemographic characteristics and clinical information, including age, sex, education, marital status, employment, living arrangement, history of chronic disease, etc. Anthropometric indices (body weight, height, waist circumference) were obtained at the same visit and used to derive BMI.

At each assessment time point, psychological distress was measured using the Distress Thermometer, and psychosocial factors potentially associated with distress trajectories were assessed using validated instruments, including perceived social support (PSSS), illness perception (BIPQ), and coping style (SCSQ). Questionnaires were completed by participants using standardized instructions, with trained research staff available to clarify items when required, while avoiding leading prompts.

To ensure data quality, all assessors received uniform training before study initiation. Completed questionnaires were checked for completeness at the point of collection, and data were double-entered and cross-validated to minimize transcription errors. Participants who missed a scheduled follow-up assessment were contacted to complete the assessment within a predefined time window whenever feasible.

### 2.6 Statistical analysis

All analyses were prespecified and conducted using SPSS (version 25.0) and Mplus (version 8.3). Continuous variables were summarized as mean (SD) when approximately normally distributed and as median (IQR) otherwise. Categorical variables were summarized as number (percentage). Group comparisons were performed using analysis of variance (ANOVA) for normally distributed continuous variables, and the Mann–Whitney U test for non-normally distributed continuous variables. Categorical variables were compared using the χ² test or Fisher’s exact test as appropriate. All tests were two-sided, with P<0.05 considered statistically significant unless otherwise stated.

To identify heterogeneous developmental trajectories of psychological distress, we fitted latent class growth models (LCGM) in Mplus using repeated distress measurements at baseline and 1, 3, and 6 months. For latent class growth modeling (LCGM) performed in Mplus 8.3.

Model structure: We adopted a linear growth model to fit changes in psychological distress scores across four time points (T1–T4); nonlinear terms (quadratic/cubic) were tested but showed no significant improvement in model fit, thus excluded in the final model.

Sensitivity analysis via random resampling: To test the stability of the latent class classification, we performed repeated random resampling. Specifically, we randomly extracted 80% of the complete-case sample (n=204) without replacement for resampling and refitted the LCGM model in each resample. The resampling procedure was repeated 20 times (a standard number for resampling sensitivity tests in LCGM research). We compared model fit indices (AIC, BIC, aBIC, entropy), latent class number, class proportion, trajectory slope values and posterior probabilities across all resampled models and the primary model.

The three-class trajectory division, class proportion and trend characteristics remained consistent with the primary analysis, verifying the stability of the three-class classification solution. Model selection was guided by standard fit indices and classification diagnostics, including Akaike information criterion (AIC), Bayesian information criterion (BIC), and sample-size adjusted BIC (aBIC) (lower values indicating better fit), and entropy (values closer to 1 indicating higher classification accuracy). Competing k-class solutions were compared using the bootstrap likelihood ratio test (BLRT) and the Lo–Mendell–Rubin likelihood ratio test (LMR-LRT); a P<0.05 suggested that the k-class model fit better than the (k−1)-class model. In addition to statistical criteria, we considered interpretability and adequate class size when selecting the final trajectory solution. The dataset from the T4 (n=204) period was used for LCGM.

## 3 Results

### 3.1 Participant flow and baseline characteristics

A total of 234 participants were included in this study. Baseline data were collected at T1. After one month of weight loss, 11 participants were excluded, and data from 223 participants were collected at T2. After three months of weight loss, 9 participants were lost to follow-up, and data from 214 participants were collected at T3. After six months of weight loss, 10 participants were lost to follow-up, and data from 204 participants were collected at T4 (**Figure 1**).

**Figure 1:**
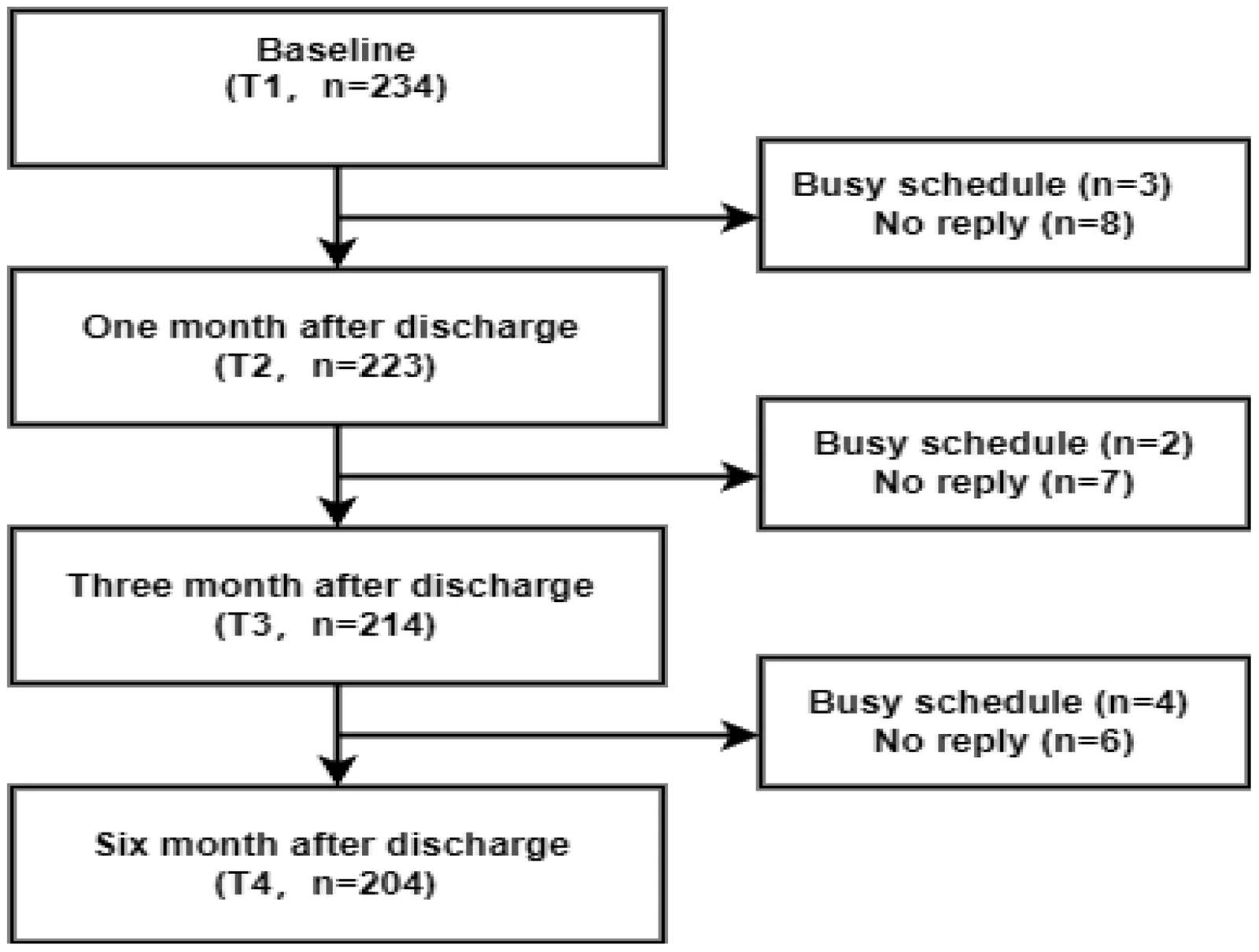
Participant flow and follow-up assessments.

We compared baseline characteristics between participants retained in the cohort (n=204) and those lost to follow-up (n=30). No statistically significant differences were observed across demographic, anthropometric, clinical, or weight-loss related variables (all P>0.05), suggesting limited evidence of attrition-related selection bias (**Table 1**). The final analytic sample (n=204) refers to enrolled participants who completed all four waves of assessments.204 participants completed 6-month body weight measurement. The overall mean percentage of body weight loss was 8.36% (SD = 2.15). Subgroup results by weight-loss method: bariatric surgery group (12.17%, SD = 1.89), anti-obesity medication group (8.92%, SD = 1.76), lifestyle intervention group (5.41%, SD = 1.63). One-way ANOVA confirmed significant differences in weight loss magnitude among the three groups (F = 226.452, P<0.001).

**Table 1:** Baseline characteristics of participants retained in the cohort and those lost to follow-up.

Variables	Analytic sample	Not included in final	Test statistic	P value
	(n=204)	analysis (n=30)		
Ages (years), M±SD	32.70±10.02	32.62±9.84	t=0.04	0.968
BMI (kg/m2), M±SD	35.24±5.54	36.01±5.72	t=-0.708	0.478
Sex, n (%)			χ2=0.107	0.744
Male	82(40.20)	13(43.33)		
Female	122(59.80)	17(56.67)		
Marital status, n (%)			χ2=0.132	0.716
Others	88(43.13)	14(46.67)		
Married	116(56.86)	16(53.33)		
Employment status, n			χ2=0.322	0.570
(%)				
Unemployed	79(38.73)	10(33.33)		
Employed	125(61.27)	20(66.67)		
Educational background, n (%)			χ2=1.278*	0.734
High school and below	24(11.76)	3(10.00)		
College degree	49(24.02)	7(23.33)		
Bachelor's degree	94(46.08)	12(40.00)		
Master's degree	37(18.14)	8(26.67)		
Chronic disease, n			χ2=0.105	0.746

NRCMS, New Rural Cooperative Medical Scheme; URBMI, Urban Residents.

Basic Medical Insurance; UEBMI, Urban Employees Basic Medical Insurance; *Fisher’s exact test. DT, Distress Thermometer.

### 3.2 Psychological distress Prevalence over follow-up

Psychological distress (DT ≥4) was common at the initial clinic visit, affecting 44.12% of participants (90/204). The prevalence increased at 1 month to 52.45% (107/204), and then declined at 3 months (40.20%, 82/204) and 6 months (36.27%, 74/204). Mean (SD) DT scores at baseline, 1 month, 3 months, and 6 months were 5.19 (0.84), 6.19 (1.42), 5.24 (1.37), and 4.87 (1.06), respectively. Corresponding 95% CIs were 5.04-5.35, 6.05-6.34, 5.15-5.44, and 4.65-5.09. Overall, distress scores increased during the first month after treatment initiation and subsequently declined over follow- up (**Figure 2**). Pairwise comparisons across time points demonstrated statistically significant differences in distress levels over time (**Table 2**).

**Figure 2:**
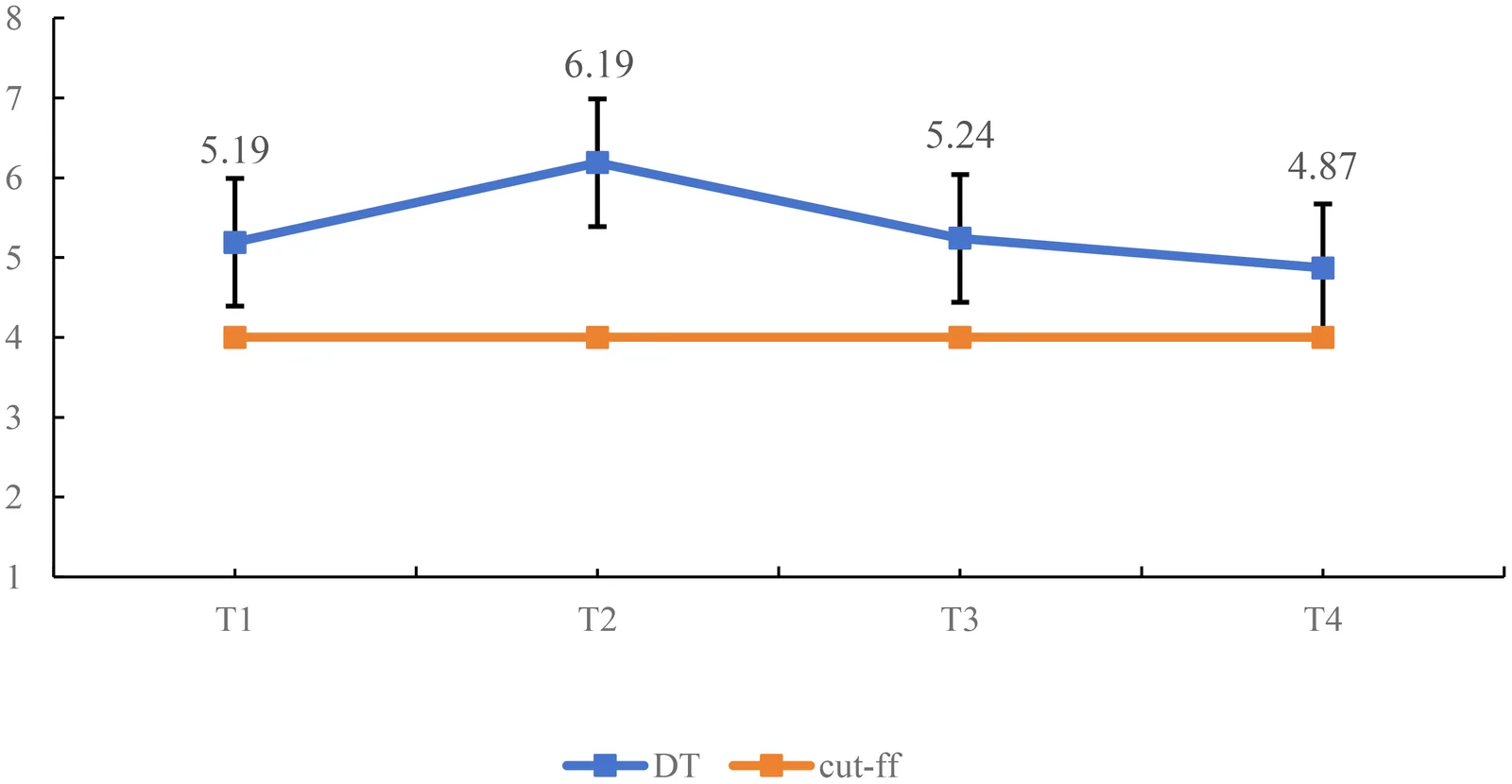
The overall trend of psychological distress among adults with obesity.

**Table 2:** Multiple comparisons of psychological distress scores in adults with obesity.

Time	Time	Marginal mean difference	P
T1	T2	-0.995 (95%CI -1.275∼-0.715)	<0.001
	T3	-0.044 (95%CI -0.359∼-0.163)	1.000
	T4	0.324 (95%CI -0.015∼-0.662)	0.069
T2	T3 T4	0.951 (95%CI -0.648∼1.146) 1.319 (95%CI 0.942∼1.695)	<0.001 <0.001
T3	T4	0.368 (95%CI 0.174∼-0.669)	<0.001

### 3.3 Identification of latent distress trajectories

Model fit indices for LCGM solutions with one to four classes are presented in **Table 3**. In the LCGM, the 3-class solution showed strong classification quality (entropy=0.903) and both the LMRT and BLRT were statistically significant; in contrast, the 4-class LCGM had higher entropy (0.934) but a non-significant LMR-LRT, suggesting limited incremental improvement. Considering statistical fit, classification, and interpretability, we selected the three-class LCGM solution. The estimated trajectories are shown in **Figure 3**. Trajectory 1 (C1) reflected decreasing distress (n=130 [63.7%]; slope, -0.584; 95% CI, -0.714 to -0.435; P<0.001; average posterior probability, 0.923); trajectory 2 (C2) reflected increasing distress (n=43 [21.1%]; slope, 0.932; 95% CI, 0.783 to 1.088; P<0.001; average posterior probability, 0.876); and trajectory 3 (C3) reflected persistently no/low distress (n=31 [15.2%]; slope, -0.061; 95% CI, -0.153 to 0.037; P = 0.215; average posterior probability, 0.857).

**Table 3:** Model fit indices for LCGM of psychological distress trajectories (n=204).

Model	No. of classes	k	LL	AIC	BIC	aBIC	Entropy	P (LMRT)	P (BLRT)	Proportion
LCGM	1	6	-1423.367	2858.735	2878.643	2859.634	—	—	—	1.000
	2	8	-1422.853	2861.707	2888.251	2862.905	0.887	0.054	0.065	0.819/0.181
	3	10	-1403.401	2826.802	2859.983	2828.300	0.903	<0.001	<0.001	0.637/0.211/0.152
	4	12	-1381.894	2787.788	2827.605	2789.585	0.934	0.231	<0.001	0.151/0.274/0.404/0.171

AIC, Akaike information criterion; BIC, Bayesian information criterion; aBIC, sample-size adjusted.

BIC; BLRT, bootstrap likelihood ratio test; LCGM, latent class growth model; LMR-LRT, Lo–Mendell–Rubin likelihood ratio test.

Lower AIC/BIC/aBIC indicates better fit. Entropy ranges from 0 to 1, with values closer to 1 indicating better classification. LMR-LRT and BLRT P values compare the k-class model with the (k–1)-class model. Class proportions are listed in the order of classes (C1 to Ck).

**Figure 3:**
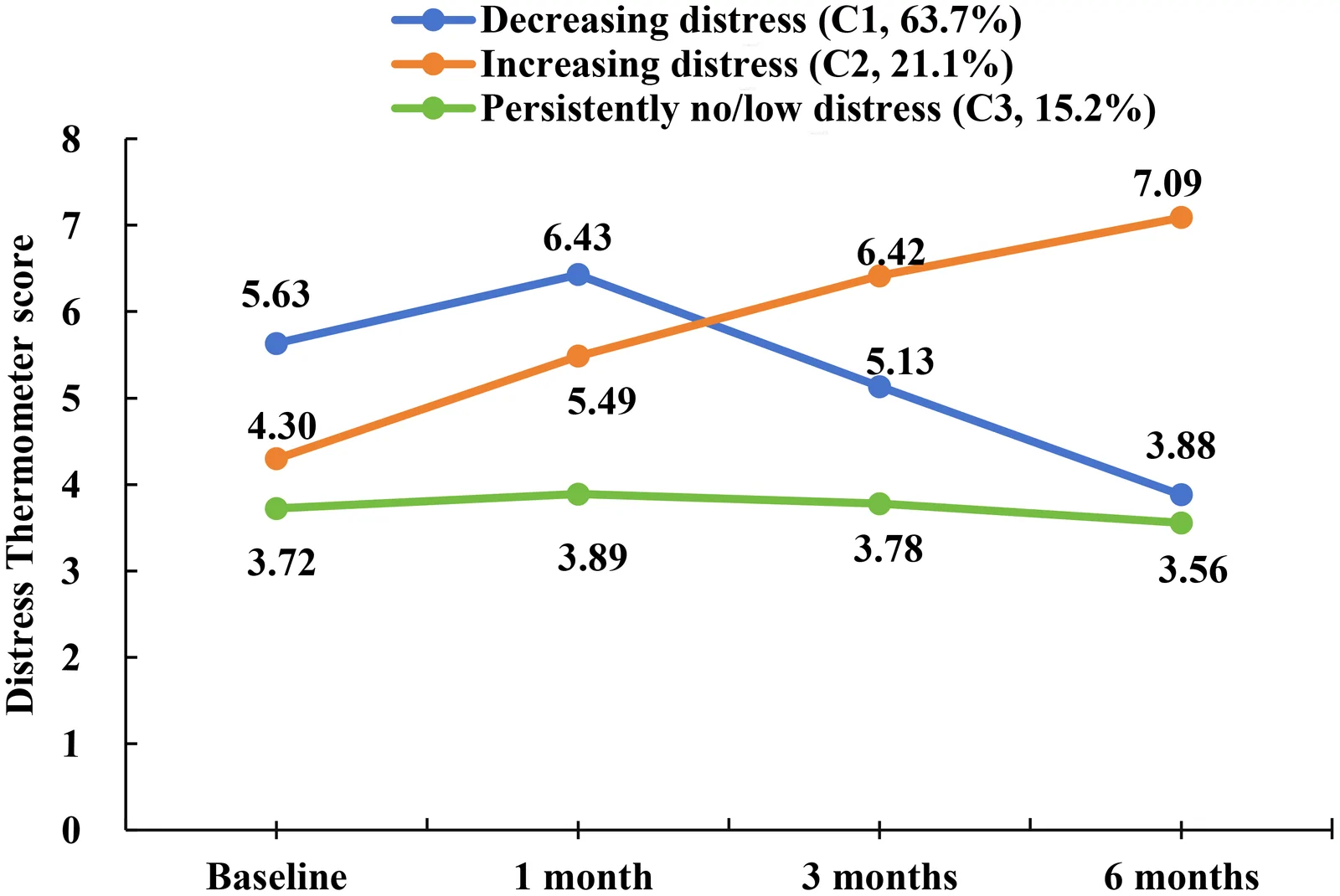
Latent trajectories of psychological distress during weight loss identified by latent class growth modelling (LCGM) (n=204).

Results of resampling sensitivity analysis: Across all 20 resampling runs, all resampled models consistently selected the 3-class solution as the optimal model. The range of entropy values across resamples was 0.891–0.916, which was close to the primary model entropy (0.903). The relative proportion of the three trajectories, linear slope coefficients and corresponding statistical significance showed no substantial deviations from the primary analysis. No model shift to 2-class or 4-class solutions occurred in any resample. These findings fully confirm that the three-distress-trajectory classification of the primary LCGM model is statistically stable and reliable.

### 3.4 Univariable associations with distress-trajectory membership

Baseline characteristics and psychosocial measures were compared across the three latent distress trajectories (decreasing distress, increasing distress, and persistently no/low distress). In univariable analyses, trajectory membership was associated with age, sex, BMI, and Chronic disease ,PSSS ,BIPQ ,SCSQ have statistical significance (all P<0.05). Participants in the increasing-distress trajectory tended to have higher BMI, lower perceived social support, more negative illness perceptions, and greater reliance on negative coping strategies at baseline than those in the decreasing-distress or persistently no/low distress trajectories (all P<0.05).

By contrast, variables such as marital status, educational attainment, employment, living arrangement, medical expense coverage, monthly income, chronic disease history, and weight-loss method showed no clear evidence of association with trajectory membership in univariable comparisons (all P>0.05). Detailed results are presented in **Table 4**.

**Table 4:** Univariable associations with psychological distress trajectory membership during the first 6 months of weight loss among adults with obesity (n=204).

Variables	C1 (n=130)	C2 (n=43)	C3 (n=31)	χ2/F	P Value
Age (years), M±SD	31.05±9.76	31.58±8.70	36.12±9.43	3.611b	0.025
Sex, n (%)								
Male	48(36.92)	15(34.88)	19(61.29)	6.822a	0.033
Female	82(63.08)	28(65.12)	12(38.71)		
Marital status, n (%)								
Others	55(42.31)	22(51.16)	11(35.49)	1.906a	0.386
Married	75(57.69)	21(48.84)	20(64.51)		
BMI (kg/m2), M±SD	38.66±5.37	34.39±3.57	32.09±6.04	22.752b	0.001
Waist, circumference, (cm)	110.56±9.72	107.46±10.43	104.93±12.00	4.432b	0.002
Educational attainment,n (%)								
High school or below	12(9.23)	6(13.96)	6(19.35)	5.894a	0.435
College	34(26.16)	8(18.60)	7(22.58)		
Bachelor's	64(49.23)	18(41.86)	12(38.71)		
Master's	20(15.38)	11(25.58)	6(19.35)		
Employment status, n (%)								
Unemployed	49(37.69)	16(37.21)	14(45.16)	0.641a	0.726
Employed	81(62.31)	27(62.79)	17(54.845)		
Chronic disease, n (%)								
No	46(35.38)	24(55.81)	18(58.06)	8.819a	0.012
Yes	84(64.62)	19(44.19)	13(41.94)		
Per capita monthly income (RMB), n (%)								
<3000	36(27.69)	11(25.58)	10(32.26)	1.194a	0.879
3000∼6000	58(44.62)	17(39.53)	12(38.71)		
>6000	36(27.69)	15(34.89)	9(29.03)		
Primary insurance status, n (%)								
No	14(10.77)	9(20.93)	7(22.58)	8.889a	0.180
NRCMS	42(32.31)	11(25.58)	10(32.26)		
URBMI	59(45.38)	15(34.89)	8(25.81)		
UEBMI	15(11.54)	8(18.60)	6(19.35)		
Weight loss goal, n (%)		15.004a	0.004		
<5%	17(13.08)	11(25.58)	12(38.71)		
5%∼10%	32(24.62)	13(30.23)	9(29.03)		
11%∼15%	81(62.30)	19(44.19)	10(32.26)		
Weight loss method, n (%)				10.856a	0.0028
Metabolic/bariatric surgery	21(16.16)	9(20.93)	13(41.94)		
Anti-obesity drugs	48(36.92)	12(27.91)	8(25.80)		
Diet and exercise	61(46.9)	22(51.16)	10(32.26)		
Living arrangement, n (%)				6.349a	0.175
Live alone	26(20.00)	8(18.60)	8(25.81)		
Dormitory	17(13.08)	8(18.60)	9(29.03)		
Living with family	87(66.92)	27(62.80)	14(45.16)		
PSSS, M±SD	37.44±9.38	39.04±6.82	51.77±5.68	36.492b	P<0.001
BIPQ, M±SD	54.64±10.77	47.49±12.64	44.68±15.33	11.968b	P<0.001
SCSQ, M±SD								
Positive	3.92±6.42	15.79±5.96	19.74±5.75	11.491b	P<0.001
Negative	20.11±3.64	19.19±3.43	16.41±3.97	12.886b	P<0.001

BIPQ, Brief Illness Perception Questionnaire; BMI, body mass index; DT, Distress Thermometer; PSSS, Perceived Social Support Scale; SCSQ, Simplified Coping Style Questionnaire; NRCMS, New Rural Cooperative Medical Scheme; URBMI, Urban Residents Basic Medical Insurance; UEBMI, Urban Employees Basic Medical Insurance.

Test statistics are χ² for categorical variables and ANOVA F for continuous variables. C1: decreasing distress trajectory; C2: increasing distress trajectory; C3: consistently no/low distress trajectory.

### 3.5 Multivariable analysis of distress-trajectory membership

We fitted a multinomial logistic regression model to identify baseline factors independently associated with distress-trajectory membership, using the persistently no/low distress group (C3) as the reference category. Overall model fit was statistically significant (χ²=78.035; P<0.001), indicating that the included predictors jointly differentiated between trajectory classes.

Compared with C3, membership in the decreasing-distress trajectory (C1) was associated with lower age (OR, 0.858; 95% CI, 0.745 to 0.988; P = 0.034), presence of chronic disease (OR, 1.293; 95% CI, 1.018 to 1.642; P = 0.035), higher negative coping (SCSQ-negative: OR, 1.445; 95% CI, 1.166 to 1.791; P = 0.001), lower perceived social support (PSSS: OR, 0.912; 95% CI, 0.832 to 1.000; P = 0.049), and more negative illness perceptions (BIPQ: OR, 1.064; 95% CI, 1.002 to 1.163; P = 0.044).

Compared with C3, membership in the increasing-distress trajectory (C2) was associated with lower age (OR, 0.827; 95% CI, 0.760 to 0.988; P = 0.033), sex (male vs female: OR, 0.131; 95% CI, 0.024 to 0.708; P = 0.018), higher BMI (OR, 1.337; 95% CI, 1.055 to 1.695; P = 0.016), higher negative coping (SCSQ-negative: OR, 1.319; 95% CI, 1.043 to 1.667; P = 0.021), lower perceived social support (PSSS: OR, 0.867; 95% CI, 0.793 to 0.947; P = 0.002), and more negative illness perceptions (BIPQ: OR, 1.080; 95% CI, 1.002 to 1.164; P = 0.043). Full model estimates are provided in **Table 5**.To evaluate the stability of the multinomial regression model, we conducted sensitivity analysis: we sequentially removed one predictor each time and refitted the model. The direction, magnitude and statistical significance of odds ratios (ORs) for all core predictors did not change substantially, indicating that the regression results were relatively stable and the overfitting risk was low.

**Table 5:** Multinomial logistic regression identifying baseline predictors of psychological distress trajectory membership during weight loss (n=204).

Class	Predictor	B	SE	Wald χ2	P	OR	95%CI	
							Lower	Upper
C1 vs C3	Constant term	-3.065	7.147	0.184	0.668	–	–	–
	Age	-0.153	0.072	4.499	0.034	0.858	0.745	0.988
	Chronic							
	diseases							
	Yes	0.257	0.122	4.446	0.035	1.293	1.018	1.642
	No	reference						
	SCSQ							
	Negative	0.368	0.109	11.334	0.001	1.445	1.166	1.791
	PSSS	-0.092	0.047	3.878	0.049	0.912	0.832	1.000
	BIPQ	0.077	0.038	4.040	0.044	1.064	1.002	1.163
C2 vs C3	Constant term	-2.754	6.640	0.172	0.678	–	–	–
	Age	-0.143	0.067	4.564	0.033	0.827	0.760	0.988
	Sex							
	Male	-2.033	0.861	5.575	0.018	0.131	0.024	0.708
	Female	reference						
	BMI SCSQ Negative	0.291 0.277	0.121 0.120	5.772 5.364	0.016 0.021	1.337 1.319	1.055 1.043	1.695 1.667
	PSSS	-0.143	0.045	9.978	0.002	0.867	0.793	0.947
	BIPQ	0.077	0.038	4.091	0.043	1.080	1.002	1.164

BMI, body mass index; BIPQ, Brief Illness Perception Questionnaire; PSSS, Perceived Social Support Scale; SCSQ, Simplified Coping Style Questionnaire.

C1: decreasing distress trajectory; C2: increasing distress trajectory; C3: persistently no/low distress trajectory (reference). Continuous predictors were entered as continuous variables (per 1- unit increase). For categorical predictors, the reference categories were no chronic disease and female. Model fit: χ²=78.035, P<0.001.

## 4 Discussion

### 4.1 The trajectories of psychological distress during weight loss among adults with obesity

In this prospective cohort, latent class growth modeling identified three distinct trajectories of psychological distress over the first 6 months of weight loss: a decreasing distress trajectory (C1, 63.7%), an increasing distress trajectory (C2, 21.1%), and a persistently no/low distress trajectory (C3, 15.2%). These findings underscore clinically meaningful heterogeneity in psychological responses during early weight management, rather than a uniform pattern of improvement (4). The predominance of the decreasing trajectory suggests that distress is frequently elevated around the initiation of weight loss, but tends to abate for many individuals as treatment engagement stabilizes and early behavioral routines become consolidated, consistent with prior reports that psychosocial well-being may improve after sustained weight-loss efforts and/or metabolic outcomes improve (16, 17).

The decreasing-distress pattern is plausibly explained by the high psychological demands of the early phase of weight loss, when patients confront restrictive diet changes, increased self-monitoring, and uncertainty about whether efforts will yield visible results (18). As weight loss progresses, improvements in weight-related symptoms and perceived control, alongside increased reinforcement from family, peers, and clinicians, may contribute to a gradual reduction in distress (7, 19). Longitudinal evidence among bariatric surgery cohorts also suggests that distress often declines after the acute preoperative period and early postoperative adaptation, potentially reflecting both physiological improvement and strengthened treatment-related support structures (20).

In contrast to studies restricted to metabolic/bariatric surgery candidates, our cohort included individuals undergoing heterogeneous weight-loss approaches (lifestyle intervention, pharmacotherapy, and surgery), which may entail varying degrees of treatment burden, adverse effects, and uncertainty in short-term outcomes; these factors could shift the distribution of low-distress trajectories (21). This discrepancy is likely attributable to differences in case mix and intervention context. In contrast to studies restricted to metabolic/bariatric surgery candidates, our cohort included individuals undergoing heterogeneous weight-loss approaches (lifestyle intervention, pharmacotherapy, and surgery), which may entail varying degrees of treatment burden, adverse effects, and uncertainty in short-term outcomes; these factors could shift the distribution of low-distress trajectories (22).

A clinically important finding is that approximately one-fifth of patients (21.1%) followed an increasing-distress trajectory, aligning with epidemiological evidence showing substantial rates of psychological distress among individuals with obesity (23). This subgroup warrants particular clinical attention because worsening distress during weight loss may compromise adherence, increase emotional eating, and undermine sustained behavior change. The higher representation of younger women in the increasing-distress group in our cohort parallels prior observations that female sex and younger age are associated with heightened vulnerability to distress in obesity-related contexts, potentially reflecting stronger appearance-related concerns, greater sensitivity to short-term outcome fluctuations, and competing work-family demands that impede consistent self-management (18).

### 4.2 Predictors of psychological-distress trajectories during weight loss

In our cohort, distress-trajectory membership was not random; it clustered with a set of baseline demographic, clinical, and psychosocial characteristics. Younger women were more likely to follow an increasing-distress trajectory during the first 6 months of weight loss, broadly aligning with prior evidence that younger female patients may experience greater appearance- and outcome-related pressure during weight management (24). Plausible mechanisms include heightened sensitivity to short-term changes in body shape and perceived “lack of progress”, internalized weight stigma, and avoidance of social interaction, all of which can amplify anxiety and worry in the absence of early visible results (25). In addition, young adults often carry competing family and work demands, which may erode time and cognitive bandwidth for sustained self-management and increase frustration when lifestyle changes conflict with daily responsibilities (26, 27). Clinically, these findings support proactive monitoring for early distress escalation in young women, with anticipatory counselling that aligns expectations about the pace of change, provides rapid-access support when setbacks occur, and integrates brief, structured psychological check-ins during the first 1 to 3 months of treatment (28).

Perceived social support also emerged as a key correlate of trajectory membership. Higher support was more common among individuals in the persistently no/low- distress trajectory, consistent with prior work demonstrating the buffering role of family, peer, and workplace support in sustaining behavior change (29). Weight loss is typically prolonged, resource-intensive, and vulnerable to discontinuity; when support is limited—especially after early goals are not met—patients may experience marked frustration and psychological pain, undermining adherence and reinforcing distress (30). From an intervention standpoint, routine assessment of social support could be used to triage patients to family-involved counselling, peer-group programs, or structured follow-up (including digital touchpoints) to reduce isolation and strengthen persistence during periods of slow progress (31).

Illness perceptions further differentiated trajectories. More negative perceptions of obesity — reflecting beliefs that the condition is severe, uncontrollable, or personally threatening—were associated with greater distress risk in our cohort. Such illness representations can shape emotional responses and coping choices, potentially sustaining anxiety and depressive symptoms even when objective weight outcomes improve (32). Evidence from population studies suggests that more negative weight- and health-related perceptions track with higher obesity-related risk and may reinforce maladaptive appraisal processes (33). These findings underscore the clinical value of addressing illness perceptions directly (e.g., through education, collaborative goal-setting, and cognitive reframing) as part of comprehensive weight management, rather than treating distress as an isolated comorbidity (34).

Finally, coping style appeared closely linked to distress trajectories. Active coping was more consistent with a decreasing-distress pattern, whereas reliance on negative coping strategies aligned with less favorable trajectories. This is coherent with the construct assessed by the Simplified Coping Style Questionnaire, which captures adaptive versus maladaptive responses to stress and setbacks (35). Patients who employ active coping may be better able to tolerate short-term discomfort, problem-solve barriers, and maintain engagement with dietary and physical-activity plans. For those with chronic comorbidity, structured behavioral change may also generate tangible improvements in cardiometabolic markers, reinforcing self-efficacy and emotional relief (36). Conversely, passive coping may contribute to inconsistent implementation of weight-management plans, weight rebound, and persistent distress escalation over time (37). Taken together, these results support embedding coping-skills guidance into routine care—helping patients plan realistic schedules, negotiate work-family-treatment conflicts, and set achievable, staged weight-loss targets—to reduce avoidable distress and improve the sustainability of behavior change (38).

### 4.3 Limitations

Several limitations should be considered when interpreting these findings. First, this was a single-center prospective cohort conducted in a tertiary hospital weight- management clinic in Jiangxi Province. Although the center receives a large volume of referrals and may capture a broad spectrum of obesity severity in the region, selection bias cannot be excluded, and the external generalizability of both the trajectory patterns and the prediction model to other settings (e.g., primary care, community programs, or centers with different treatment pathways) remains uncertain.

Second, follow-up was limited to 6 months, which reflects early adaptation to weight-loss treatment but may not capture longer-term distress trajectories, including relapse, weight regain, and delayed psychological responses. Longer follow-up with repeated measures is needed to clarify whether the identified trajectories persist, converge, or transition over time and to determine their prognostic relevance for sustained weight outcomes.

Third, psychological distress was assessed using a self-reported instrument, which is vulnerable to reporting and social-desirability bias; moreover, distress may fluctuate in response to concurrent life events that were not measured in this study. Residual confounding is therefore possible, and future studies incorporating clinician- administered assessments or multimodal measures, alongside richer time-varying covariates, would strengthen causal interpretation.

## 5 Conclusion

In this longitudinal cohort of adults with obesity, psychological distress during the first 6 months of weight loss was heterogeneous, following three distinct trajectories: decreasing distress, increasing distress, and persistently no/low distress. A clinically important subgroup showed worsening distress over time, highlighting the need for early identification rather than relying on single time-point assessment. Baseline demographic, clinical, and psychosocial factors—including age, sex, BMI, perceived social support, illness perceptions, coping style, and chronic comorbidity — were associated with trajectory membership, and an internally validated nomogram demonstrated good discrimination and calibration for risk stratification. These findings support integrating routine distress screening and targeted, scalable psychosocial support into early weight-management care, particularly within the first 1–3 months, to mitigate distress escalation and improve adherence to weight-loss programs. Future multicenter studies with longer follow-up should externally validate the prediction model and evaluate whether trajectory-informed interventions improve both psychological and weight-related outcomes.

## Data Availability

The original contributions presented in the study are included in the article/supplementary material. Further inquiries can be directed to the corresponding author.
