# Correction: Trajectories and predictors of psychological distress during the first 6 months of weight loss among adults with obesity: a prospective longitudinal cohort study

**DOI:** 10.3389/fpsyt.2026.2022923

**Published:** 2026-10-28

**Authors:** Zhiqiang Cheng, Jiaoyun Xia, Jincheng Liu, Baozhen Zhang, Jinming Liu

**Affiliations:** 1 Department of Nursing, The First Affiliated Hospital of Nanchang University, Nanchang, China; 2 School of Nursing, Jiangxi Medical College, Nanchang University, Nanchang, China; 3 The First Clinical Medical College, Gannan Medical University, Ganzhou, China; 4 Department of Cardiovascular Internal Medicine, The First Affiliated Hospital of Gannan Medical University, Ganzhou, China

**Keywords:** obesity, psychological distress, risk factors, trajectory, weight loss

In the published article, there were errors in the **Affiliations**. The affiliations were erroneously given as:

Zhiqiang Cheng^1,3^, Jiaoyun Xia^1^, Jincheng Liu^2^, Baozhen Zhang^1^ and Jinming Liu^2*^

^1^Department of Nursing, The First Affiliated Hospital of Nanchang University, Nanchang, China, ^2^Department of Cardiovascular Medicine, First Affiliated Hospital of Gannan Medical University, Nanchang, China, ^3^School of Nursing, Jiangxi Medical College, Nanchang University, Nanchang, China

The correct affiliations are:

Zhiqiang Cheng^1,2^, Jiaoyun Xia^1^, Jincheng Liu^3^, Baozhen Zhang^1^ and Jinming Liu^4*^

^1^Department of Nursing, The First Affiliated Hospital of Nanchang University, Nanchang, China, ^2^School of Nursing, Jiangxi Medical College, Nanchang University, Nanchang, China, ^3^The First Clinical Medical College, Gannan Medical University, Ganzhou, China, ^4^Department of Cardiovascular Internal Medicine, The First Affiliated Hospital of Gannan Medical University, Ganzhou, China

The original version of this article has been updated.

